# Identifying unmet needs of dementia caregivers managing dysphagia

**DOI:** 10.1093/geroni/igaf122.3180

**Published:** 2025-12-31

**Authors:** Matthew Zuraw, Zayn Boustani, Rachel Radspinner, Himalaya Patel, Teresa Thuemling, Nicole Rogus-Pulia, Nicole Werner

**Affiliations:** Better Brains, Inc, San Diego, California, United States; Indiana University Bloomington, Chicago, Illinois, United States; Better Brains, Inc, San Diego, California, United States; Indiana University Bloomington, Bloomington, Indiana, United States; Vanderbilt University Medical Center, Nashville, Tennessee, United States; University of Wisconsin–Madison School of Medicine and Public Health, Madison, Wisconsin, United States; Vanderbilt University Medical Center, Nashville, Tennessee, United States

## Abstract

The estimated prevalence of dysphagia (swallowing dysfunction) is as high as 85.9% for the more than 6 million Americans living with dementia. Dementia caregivers face significant challenges in managing dysphagia, contributing to suboptimal outcomes. Successfully intervention development requires identifying the unmet needs of dementia caregivers managing dysphagia. We conducted a needs assessment using a virtual contextual inquiry method with n = 19 dementia caregivers managing dysphagia to identify unmet needs. Virtual contextual inquiry occurred in three stages: 1) initial virtual interview focused on general dysphagia management tasks, barriers, and strategies; 2) seven days in which we prompted participants twice daily via text to send videos, images, or audio/text descriptions of daily dysphagia management activities and perform an evening reflection; 3) final Zoom interview Zoom to discuss additional context of the daily messages. We analyzed transcripts and messages using team-based analysis to identify categories of unmet needs. We identified 14 categories of unmet needs: the progression of swallowing challenges with dysphagia, how to transition from one stage of dysphagia to another, understanding when to use alternative hydration and nutrition, lack of confidence around negative outcomes, getting trustworthy information from others, ensuring successful mealtimes, what tools to use to support dysphagia, finding trustworthy knowledge, managing costs associated with dysphagia, ensuring quality of life for the person being cared for, getting proper support from professionals, handling emergencies, ensuring adequate nutrition, and managing specific dietary needs. Findings highlight the necessity for targeted resources and interventions to support dementia caregivers in dysphagia management to reduce their burden.

